# Long-Term Outcomes of Combined Medial Unicompartmental Knee Replacement and Anterior Cruciate Ligament Reconstruction in Middle-Aged Patients with ACL-Deficient Knees

**DOI:** 10.3390/jcm14186439

**Published:** 2025-09-12

**Authors:** Matteo Marullo, Stefano Petrillo, Antonio Russo, Fabrizio Bertelle, Sergio Romagnoli

**Affiliations:** 1 Department of Joint Replacement, IRCCS Istituto Ortopedico Galeazzi, 20157 Milan, Italy; 2 Humanitas Torino, 10126 Turin, Italy

**Keywords:** unicompartmental knee replacement, anterior cruciate ligament, knee osteoarthritis, ACL deficiency, small implants

## Abstract

**Background**: Successful unicompartmental knee arthroplasty (UKA) requires complete ligamentous competence, including the anterior cruciate ligament (ACL). The present study evaluated the long-term outcomes, complications, survival, and osteoarthritis (OA) progression in patients with medial femorotibial OA and ACL lesions undergoing simultaneous combined UKA and ACL reconstruction (ACLR). **Methods**: Patients who underwent simultaneous medial UKA and ACLR or revision ACLR from January 2004 to December 2021 were retrospectively reviewed. Inclusion criteria were a minimum follow-up period of 2 years and implantation of a cemented, fixed-bearing UKA. Outcomes were measured using the Knee Society Score (KSS), Tegner Activity Scale (TAS), University of California, Los Angeles (UCLA) Activity Score, and range of motion (ROM). **Results**: Thirty-four patients met the inclusion criteria. Mean follow-up was 11.7 years. Mean age was 52 years. Patients demonstrated significant improvements in KSS-C (from 52.8 ± 6.8 to 94.9 ± 7.9), KSS-F (from 58.3 ± 10.0 to 98.1 ± 4.2), TAS (from 0.7 ± 0.5 to 4.9 ± 1.1), UCLA (from 1.4 ± 0.6 to 6.6 ± 1.4), and ROM (from 109.1 ± 8.9 to 126.3 ± 6.1) (*p* < 0.01). Survival rate was 97.1% at 11.7 years. Lachman test results improved significantly (from 16 patients with grade II and 16 grade III to 13 grade 0 and 19 grade I, *p* < 0.01). No significant difference in functional outcomes was found between primary and revision ACLR groups; however, patients undergoing revision ACLR exhibited higher OA progression in the lateral compartment (*p* = 0.03). **Conclusions**: Simultaneous medial UKA and ACLR or revision ACLR led to excellent long-term outcomes, high survival rates, significant functional improvements, and minimal OA progression in the lateral compartment.

## 1. Introduction

The management of combined anterior cruciate ligament (ACL) deficiency and medial femorotibial osteoarthritis (OA) is challenging, especially in young, active, and athletic patients. In order to choose the right options for these patients, the primary pathology should be explored.

If arthritis is the primary problem, it tends to begin anteriorly in the medial compartment and then extend posteriorly and progressively damage the ACL [1,2]. This tends to be associated with shortening of the medial collateral ligament and progressive lateral-compartment arthritis [1]. Thus, in primary medial compartment OA with secondary rupture of the ACL, a combined anterior cruciate ligament reconstruction (ACLR) and unicompartmental knee arthroplasty (UKA) is not considered appropriate because of the other associated changes. These patients, who are usually elderly, should be considered for total knee arthroplasty (TKA).

In contrast, if the ACL deficiency is the primary problem, the posterior dislocation of the femur significantly overloads and wears down the posteromedial cartilage of the tibial plateau [3,4]. These patients tend to be relatively young and active, and the medial collateral ligament and lateral compartment are relatively normal [1,3,4]. In such situations, current surgical options include high tibial osteotomy (HTO) with or without ACLR, UKA alone or combined with ACLR, and TKA [1,2,5,6,7,8,9].

ACL damage was once considered a contraindication for HTO or UKA alone [5,6,7,8]. HTO with concurrent ACLR is effective for young patients with medial OA symptoms and ACL deficiency, restoring proper knee alignment and stability [6]; however, combined HTO and ACLR surgery shows a threefold higher complication rate compared to UKA, primarily due to graft failure [1,5,8].

For elderly patients with severe OA and ACL damage, TKA remains the preferred surgical approach [7,10]. However, TKA is less suitable for young and active patients with isolated knee OA and ACL damage. UKA offers the following advantages over TKA: bone preservation, soft-tissue sparing, rapid recovery, reduced perioperative complications, better range of motion, and more physiological function [10,11,12,13,14]. Nevertheless, using UKA in ACL-deficient knees remains controversial due to altered knee biomechanics, with reports of high poly wear in fixed-bearing implants and aseptic loosening in mobile-bearing implants [15,16].

Staged ACLR and UKA could be an option but require two surgeries, increasing complication risks and extending recovery time [17].

The rationale behind combining UKA and ACLR is to address medial OA and ACL deficiency simultaneously, reducing medial pain and restoring normal knee biomechanics [17,18].

This study aimed to evaluate outcomes, complications, survival, and OA progression in patients with medial femorotibial OA and ACL lesion undergoing simultaneous combined UKA and ACLR surgery. The hypothesis is that combined UKA + ACLR provides excellent functional outcomes and high survival rates even at long-term follow-up, with minimal or no OA progression in the lateral compartment.

## 2. Materials and Methods

The medical records of all patients who underwent simultaneous medial UKA and ACLR at our institution between January 2004 and December 2021 were retrospectively reviewed. Inclusion criteria were as follows: simultaneous medial UKA and ACLR (or revision ACLR) with a cemented, fixed-bearing, metal-backed UKA and a postoperative follow-up period of at least 2 years. Patients were excluded if they received a different prosthesis, had incomplete medical records, or the follow-up period was shorter than 2 years.

**Figure 1:**
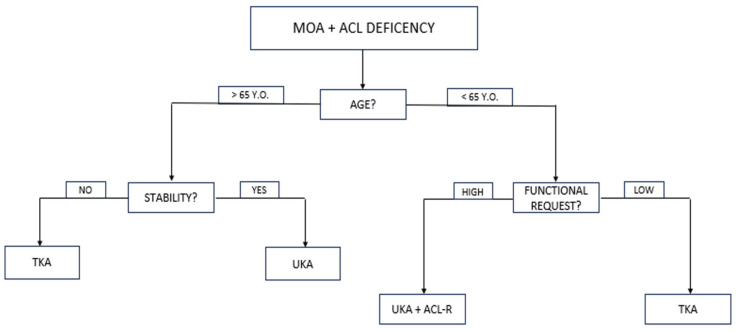
Algorithm for determining if a patient is a suitable candidate for simultaneous UKA and ACLR. MOA, medial osteoarthritis; ACL, anterior cruciate ligament; UKA, unicompartmental knee replacement; TKA, total knee replacement.

Indications for simultaneous medial UKA and ACL reconstruction were as follows: primary ACL lesion and secondary symptomatic medial tibiofemoral OA (Kellgren–Lawrence [KL] grade 3 or higher [19]); no OA in the lateral or patellofemoral compartments (KL grade 0 or 1); no lesions of the collateral ligaments; clinical and radiographic evidence of ACL deficiency; age under 65. An algorithm for determining if a patient is a suitable candidate for simultaneous UKA and ACLR is presented in Figure 1.

Contraindications for medial UKA and ACL reconstruction included the following: OA in any compartment other than the medial tibiofemoral; collateral ligament deficiency; preoperative range of motion (ROM) less than 90°; flexion contracture greater than 10°; inflammatory disease.

This study was approved by the Ethical Committee of our institution (reference number: 134/INT/2017; clinicaltrials.gov ID: NCT04198389, dated on 12 October 2017, amended on 10 March 2021) and conducted following the STROBE Checklist for Case Series [20]. Written informed consent was obtained from all participants.

### 2.1. Surgical Technique and Rehabilitation

All procedures were performed by two senior orthopedic surgeons (M.M. and S.R.). Medial UKA was performed via a standard medial parapatellar approach with a mini-midvastus arthrotomy. ACL reconstruction was performed either arthroscopically or through an open approach. In all cases, a tourniquet was not used [21], and a cemented, fixed-bearing, metal-backed UKA was implanted (Allegretto^®^, Zimmer Biomet Inc., Warsaw, IN, USA; ZUK Zimmer Unicompartmental Knee^®^, Zimmer Inc., Warsaw, IN; or Persona Partial Knee^®^, Zimmer Biomet Inc., Warsaw, IN, USA). Graft fixation was achieved using a suspension technique on the femoral side and an interference screw on the tibial side in all cases. The alignment goal after UKA was a mild undercorrection of the deformity [22]. As the medial wear in patients with ACL deficiency is located posteriorly, these patients exhibited a pronounced posterior slope. In all cases, the posterior slope of the tibial component was set to 0–3° to decrease anterior translation of the tibia and protect the ACL graft [23].

In arthroscopically assisted ACLR, ACL reconstruction was performed first. The femoral tunnel was created through the anteromedial portal, ensuring that the tibial and femoral tunnels were unlinked. This technique is advantageous in revision ACLR as it allows for anatomical graft placement while enabling differently oriented tunnels compared to the previous ones, thereby achieving adequate bone-graft contact. After placing the graft in the tunnels, it was fixed in the femur but left unfixed in the tibia. The UKA was then implanted, and after polyethylene insertion, the graft was fixed in the tibia with an interference screw.

**Figure 2:**
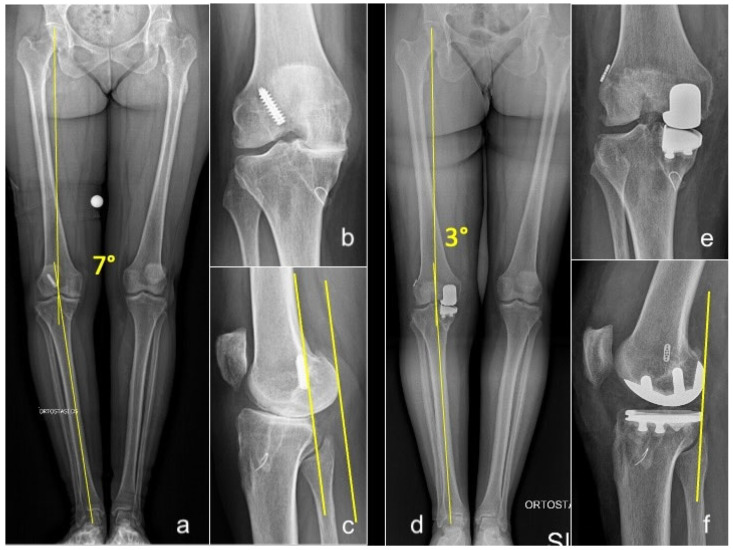
Long-standing whole-leg (**a**), anteroposterior (**b**), and lateral (**c**) radiographs of a 54-year-old female who presented with grade 4 medial OA and failure of a previous ACLR. Postoperative long-standing whole-leg (**d**), anteroposterior (**e**), and lateral (**f**) views after simultaneous medial UKA + revision of ACLR. Yellow lines in (**a**,**d**): hip-knee-ankle angle (HKA). Yellow lines in figure (**c**,**f**): posterior translation of the femoral condyles consequent to ACL deficiency, corrected after UKA+ACLR.

In open ACLR, UKA was performed first to correct the coronal deformity before placing the ACL graft. After trial reduction in the UKA, the femoral tunnel was created via the arthrotomy, and a guiding wire was passed through it. Then the UKA was cemented and, subsequently, the tibial tunnel was created to prevent cement invasion. The graft was then passed using the guiding wire and secured to the femur and tibia (Figure 2).

All patients began passive and active range of motion (ROM) exercises within 12 h post-surgery. Progressive weight-bearing commenced on postoperative day 1. Patients were discharged on the second postoperative day once they demonstrated independent walking with crutches and achieved at least 90° of knee flexion.

### 2.2. Clinical and Radiographic Evaluation

Preoperative patient characteristics included gender, age at surgery, body mass index (BMI), the degree of tibiofemoral and patellofemoral degeneration according to the Kellgren and Lawrence classification [19], preoperative knee range of motion, and pain intensity measured with a visual analogue scale (VAS). Postoperative evaluations were conducted at 3 months, 12 months, and annually thereafter. Each assessment included radiological and clinical examinations, along with a patient satisfaction questionnaire. It should be noted that the questionnaire was not validated. Therefore, the data collected should be interpreted as indicative of patient experience, providing exploratory rather than standardized measurements.

The following clinical scores were assessed during follow-up visits: the Knee Society Score (KSS) [24], both clinical and functional (KSS-C and KSS-F); the Tegner Activity Scale (TAS) [25]; and the University of California, Los Angeles (UCLA) Activity Score [26]. Laxity with anteroposterior (AP) translation was assessed clinically using the Lachman test at 30° knee flexion and compared to the contralateral knee. Routine weight-bearing anterior–posterior long-leg radiographs, Rosenberg view [27], true lateral view, and 30° patellar axial view radiographs were obtained preoperatively and at every follow-up visit. The hip–knee–ankle (HKA) angle was measured both pre- and postoperatively, defined by an angle formed by a line connecting the center of the femoral head to the center of the knee, and a second line from the center of the knee to the center of the talus. Varus alignment was defined as a mechanical axis less than 180°. Radiographs were assessed by two trained observers blinded to postoperative outcomes using a Picture Archiving and Communication System (Philips Medical Systems; Sectra-Imtec AB, Linköping, Sweden). All clinical and radiological assessments were independently conducted by two examiners (S.P. and F.B.) who were not involved in the surgical procedures.

Implant failure was defined as any subsequent surgical intervention following the index procedure.

### 2.3. Statistical Analysis

Statistical analysis was conducted using the software IBM SPSS Statistics for Mac version 26.0 (IBM Corp., Armonk, NY, USA). Parametric variables were reported as means and standard deviations or range, while non-parametric variables were described as absolute number of events and percentage. Distribution was assessed with Shapiro–Wilk test. Baseline values of KSS-C, KSS-F, ROM in flexion and extension, UCLA, TAS, and coronal alignment (HKA) were compared to those registered at final follow-up using the paired *t*-test. Baseline and last follow-up levels of the Lachman test were compared using Wilcoxon’s paired sign test. Subgroup analysis was conducted using the *t*-test for independent means to investigate whether patients who underwent revision ACLR and UKA implantation showed any difference in demographic data and functional outcomes compared to those who had UKA implantation and ACLR, and to assess if there were any significant differences in the same variables between patients operated with open or arthroscopic technique. The Mann–Whitney U test was used to compare the distribution of the Lachman test and OA in the non-operated compartment between patients who underwent revision or primary ACLR. The Kaplan–Meier curve was generated to describe survival distribution of procedures. Functional outcomes were assessed to ascertain whether there was any difference based on sex of patients. Post hoc power analysis was conducted to assess the main statistical difference between subgroups, which was the higher progression of OA in patients who underwent UKA combined with revision ACLR. Post hoc power for this specific endpoint was 41.2%. Failure was considered as any further operation after the index procedure. Statistical significance was set at *p* < 0.05 for any variable assessed.

## 3. Results

During the study period, 13,225 knee replacements were performed in our Department, of which 5131 (38.8%) were UKAs; 4895 (95.4% of all UKAs) were medial UKAs. Simultaneous medial UKA and ACL reconstruction with a cemented, fixed-bearing, metal-backed UKA was performed in 34 patients (0.7% of all medial UKAs). All patients reached the minimum 2-year clinical follow-up, and their medical records were complete.

**Figure 3:**
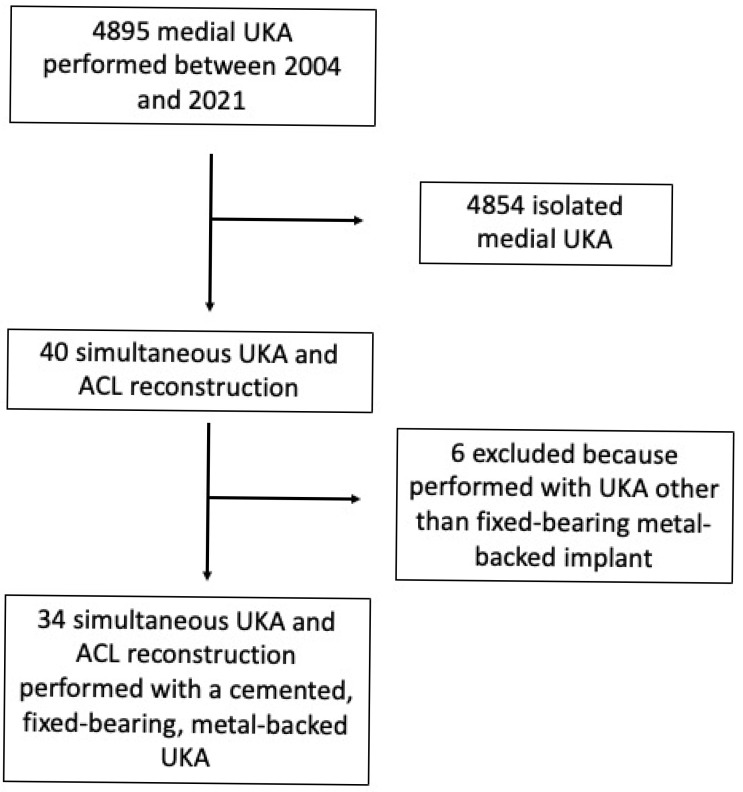
CONSORT flow chart of this study. UKA denotes unicompartmental knee arthroplasty; ACL denotes anterior cruciate ligament.

The CONSORT flow diagram is presented in Figure 3.

The study population was examined at a mean follow-up of 11.7 years (range: 2 to 21 years). The mean age was 52 years (range: 40 to 65 years), and the mean BMI was 25.8 kg/m^2^ (range: 19 to 31 kg/m^2^). Twenty-seven patients (79.4%) were men. ACLR was performed openly in twenty patients (58.8%), and arthroscopically in fourteen patients (41.2%).

In twenty patients (58.8%), ACLR was performed as primary surgery, while in fourteen patients (41.2%), it was a revision ACLR. The mean time from primary ACLR to simultaneous UKA plus revision ACLR was 15.8 years (range: 2 to 29 years).

The grafts utilized include a hamstring autograft in 2 cases (5.9%), a Ligament Advanced Reinforcement System (LARS) in 13 cases (38.2%), and an allograft (semitendinosus or tibialis anterior) in 19 cases (55.9%). Hamstring autograft was utilized in very young patients (40 and 43 years old). The LARS was used prior to 2014, mainly reflecting its extensive national use in the population over 40 years of age.

General demographic data are presented in Table 1.

### 3.1. Survival Analysis

Of the thirty-four patients included, only one (2.9%)—who belonged to the UKA + primary ACLR subgroup—ultimately underwent revision surgery 10 years after the index procedure and was converted to TKA. This corresponds to a survival rate of 97.1% at a mean follow-up of 11 years.

**Figure 4:**
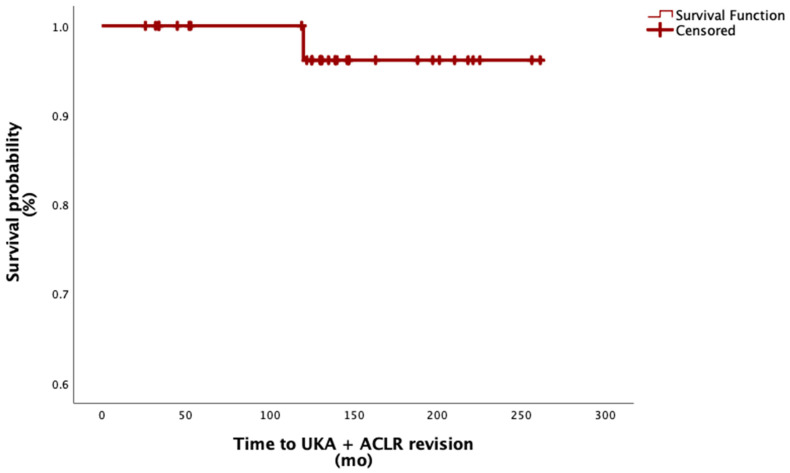
Survival function of combined UKA and ACL reconstruction. Abbreviation: mo, months.

The graft used in this patient was a hamstring autograft, which showed a survival rate of 50% in this specific graft subgroup. All other graft subgroups demonstrated a 100% survival rate at a mean follow-up of 11 years. See Figure 4.

### 3.2. Functional Outcomes

In the overall population, values for KSS-C (*p* < 0.01), KSS-F (*p* < 0.01), UCLA (*p* < 0.01), TAS (*p* < 0.01), and ROM (*p* < 0.01) significantly improved from baseline (see Table 2).

Comparing the results of patients who underwent UKA + primary ACLR and patients who underwent UKA + revision ACLR, there were no statistically significant differences in the variables mentioned above (see Table 3).

The Lachman test score significantly decreased from preoperative values to the last follow-up (from 16 patients with grade II and 16 grade III to 13 grade 0 and 19 grade I, *p* < 0.01). No significant difference was found in the Lachman test results between patients who underwent primary ACLR and those who received revision ACLR (*p* = 0.228).

Subgroup analysis based on the type of ACLR (open or arthroscopic ACLR) revealed no statistically significant differences in either demographics or functional outcomes variables (see Table 4). Male patients showed significantly higher values of postoperative KSS-C (96.3 vs. 90.0; *p* = 0.04) and KSS-F (98.8 vs. 95.4; *p* < 0.01) compared to females. However, postoperative KSS-C and KSS-F were considered excellent (>90 points) in both groups.

### 3.3. Progression of OA in the Lateral Compartment

Analysis of the progression of OA in the lateral compartment was conducted on a total of 28 patients (82.4%). In the overall population, no significant difference was found in the degree of OA in the non-operated compartment at the final follow-up compared to the preoperative status (*p* = 0.083). However, patients who underwent UKA implantation together with revision ACLR had a statistically significantly higher progression of OA in the non-operated compartment compared to those who had UKA and primary ACLR (*p* = 0.03) (see Table 5). Nevertheless, this difference did not result in an increased risk of UKA revision.

## 4. Discussion

The main finding of this paper is that simultaneous medial UKA and ACLR, including revision ACLR, results in excellent long-term outcomes for patients with ACL deficiency and subsequent medial femorotibial OA. This combined approach demonstrated a high survival rate of 97.1% at a mean follow-up of 11.7 years, significant improvements in functional scores and range of motion, and negligible progression of OA in the non-operated compartment. Both primary and revision ACLR combined with UKA were effective, with no significant differences in outcomes between the two groups.

Our findings are consistent with previous studies, reinforcing the efficacy of simultaneous medial UKA and ACLR, although long-term outcomes are limited. Existing studies are based on small groups, use non-uniform materials, and focus on medium-term follow-up. However, excellent clinical outcomes have been observed, with clinical improvement comparable to a control cohort of patients who underwent UKA with an intact ACL [18,28,29,30].

Albo et al. conducted a systematic review analyzing the outcomes of simultaneous ACLR and UKA in 169 patients with a mean follow-up of 6.3 years [17]. Their findings suggested that this combined procedure is safe and leads to improved functional and clinical outcomes, with an overall revision rate of 3.5%. However, the authors expressed concern about the potential longevity of the results [31]. Recently, Jaber et al. reported excellent 10-year outcomes in a series of 23 patients who underwent combined medial UKA and ACLR [32]. They utilized a cemented, mobile-bearing UKA, a suspension technique for graft fixation on the femoral side, and an interference screw on the tibial side. Patients showed significant improvements in knee function and pain relief, high patient satisfaction, and an implant survivorship of 91.4% at 14.5 years.

Iriberri et al. reported the long-term outcomes of eight patients who underwent UKA + ACLR [31]. They used a cemented, fixed-bearing UKA and hamstring autograft for ACLR. In this series, a patient underwent revision to TKA 9 years after the index surgery, resulting in a survival rate of 87.5% at a mean follow-up of 14.5 years.

No significant clinical and radiological differences between mobile- and fixed-bearing implant designs were found at medium-term follow-up [33].

To the best of our knowledge, our study is the first to analyze the long-term results of simultaneous medial UKA + ACLR performed with a cemented, fixed-bearing implant in a large cohort of patients.

Additionally, our study examined OA progression in the contralateral tibiofemoral compartment over time and included a substantial number of patients who had previously undergone ACLR and later underwent simultaneous UKA + revision ACLR. Thanks to these factors, our study was able to identify a higher progression of OA in the non-operated compartment among patients undergoing revision ACLR compared to primary ACLR. This suggests that revision ACLR might introduce additional biomechanical changes affecting OA progression, a topic that warrants further investigation as it was not prominently discussed in the referenced studies.

The limitations of the present study include its retrospective design and the small sample size, particularly for the UKA + revision ACLR subgroup; however, the small sample size reflects the relative rarity of this indication. Additional limitations include the absence of a control group (for example, patients treated with TKA) and the unequal distribution of graft choice among the groups, reflecting changes in graft selection practices over the study period.

## 5. Conclusions

Simultaneous medial, fixed-bearing UKA and ACLR is a viable option for managing medial knee OA with concomitant ACL deficiency. The high survival rate at long-term follow-up, significant functional improvements, and minimal OA progression in the non-operated compartment underscore the benefits of this approach. Future research should focus on larger, prospective studies to further validate these findings and explore the long-term implications of combining revision ACLR with UKA.

## Figures and Tables

**Table 1:** Demographic characteristics of the study population.

Variable	Mean ± SD	n (%)
**Gender**		
Men	–	27 (79.4)
Women	–	7 (20.6)
Age, y	52.0 ± 6.4	–
BMI, kg/m^2^	25.8 ± 2.7	–
**ACLR technique**		
Open	–	20 (58.8)
Arthroscopic	–	14 (41.2)
**Type of graft**		
Autograft	–	2 (5.9)
LARS	–	13 (38.2)
Allograft	–	19 (55.9)
Primary ACLR + UKA	–	20 (58.8)
Revision ACLR + UKA	–	14 (41.2)
Follow-up, mo	137.3 ± 64.3	–

Note. BMI = body mass index; mo = months; n = number; y = years; ACLR = anterior cruciate ligament reconstruction; UKA = unicompartmental knee arthroplasty. SD: standard deviation

**Table 2:** Clinical–functional outcomes of the overall population.

Variable	Preoperative	Final FU	*p*
ROM extension, (mean ± SD)	3.4 ± 3.0	0 ± 0	<0.001
ROM flexion, (mean ± SD)	109.1 ± 8.9	126.3 ± 6.1	<0.001
**Lachman test, n (%)**			<0.001
Grade 0	0	13 (38.2)	
Grade I	0	19 (55.9)	
Grade II	16 (47.1)	0	
Grade III	16 (47.1)	0	
KSS-C, points (mean ± SD)	52.8 ± 6.8	94.9 ± 7.9	<0.001
KSS-F, points (mean ± SD)	58.3 ± 10.0	98.1 ± 4.2	<0.001
UCLA score (mean ± SD)	1.4 ± 0.6	6.6 ± 1.4	<0.001
TAS, points (mean ± SD)	0.7 ± 0.5	4.9 ± 1.1	<0.001
HKA, ° (mean ± SD)	174.0 ± 3.7	177.8 ± 2.0	<0.001
**Lateral Kellgren–Lawrence, n (%)**			0.083
0	9 (26.5)	12 (42.9)	
1	19 (55.9)	16 (57.1)	

Note. FU = follow-up; HKA = hip–knee–ankle angle; KSS-C = Knee Society Score–Clinical; KSS-F = Knee Society Score–Function; ROM = range of motion; TAS = Tegner Activity Scale; UCLA = University of California, Los Angeles score; SD: standard deviation. *p* values are based on paired *t*-tests for continuous variables and Mann–Whitney *U* test for Lachman test.

**Table 3:** Comparison of demographic data and functional outcomes based on type of ACL reconstruction.

Variable	Primary ACLR	Revision ACLR	*p*
Age, y (mean ± SD)	53.0 ± 6.1	50.1 ± 7.0	0.26
BMI, kg/m^2^ (mean ± SD)	25.6 ± 3.2	26.7 ± 1.9	0.58
Follow-up, mo (mean ± SD)	118.5 ± 59.5	144.1 ± 55.3	0.22
**ROM flexion, (mean ± SD)**			
Preoperative	107.9 ± 9.4	110.7 ± 8.3	0.72
Last FU	127.2 ± 4.6	125.0 ± 7.6	0.036
**KSS-C, points (mean ± SD)**			
Preoperative	53.6 ± 5.4	51.8 ± 8.5	0.49
Last FU	97.5 ± 3.9	91.6 ± 10.3	0.06
**KSS-F, points (mean ± SD)**			
Preoperative	58.3 ± 13.5	58.2 ± 6.6	0.98
Last FU	99.4 ± 1.6	96.4 ± 5.7	0.08
**UCLA score (mean ± SD)**			
Preoperative	1.5 ± 0.7	1.7 ± 0.4	0.06
Last FU	6.4 ± 1.3	6.7 ± 1.5	0.60
**TAS, points (mean ± SD)**			
Preoperative	0.6 ± 0.5	0.8 ± 0.4	0.30
Last FU	4.9 ± 0.9	4.7 ± 1.4	0.71
**HKA, (mean ± SD)**			
Preoperative	173.4 ± 3.1	174.7 ± 4.3	0.38
Last FU	177.4 ± 2.2	178.3 ± 1.7	0.23

Note. ACLR = anterior cruciate ligament reconstruction; BMI = body mass index; FU = follow-up; HKA = hip–knee–ankle angle; KSS-C = Knee Society Score–Clinical; KSS-F = Knee Society Score–Function; ROM = range of motion; TAS = Tegner Activity Scale; UCLA = University of California, Los Angeles score; y = years; mo = months; SD: standard deviation. *p* values are based on independent *t*-tests comparing UKA combined with primary ACLR and UKA combined with revision ACLR.

**Table 4:** Comparison of demographic data and functional outcomes based on ACL reconstruction technique.

Variable	Open ACLR	Arthroscopic ACLR	*p*
Age, y (mean ± SD)	51.6 ± 7.8	52.1 ± 4.5	0.84
BMI, kg/m^2^ (mean ± SD)	26.4 ± 2.7	24.9 ± 2.7	0.14
Follow-up, mo (mean ± SD)	115.6 ± 67.9	150.2 ± 9.0	0.07
KSS-C, preoperative (mean ± SD)	52.9 ± 6.4	52.7 ± 7.5	0.94
KSS-C, last FU (mean ± SD)	94.2 ± 9.8	95.9 ± 4.0	0.56
KSS-F, preoperative (mean ± SD)	57.6 ± 8.6	59.2 ± 12.2	0.67
KSS-F, last FU (mean ± SD)	97.4 ± 5.1	99.2 ± 1.9	0.22

Note. ACLR = anterior cruciate ligament reconstruction; BMI = body mass index; FU = follow-up; HKA = hip–knee–ankle angle; KSS-C = Knee Society Score–Clinical; KSS-F = Knee Society Score–Function; ROM = range of motion; TAS = Tegner Activity Scale; UCLA = University of California, Los Angeles score; y = years; mo = months; SD: standard deviation. *p* values are based on independent *t*-tests comparing UKA combined with open ACLR and UKA combined with arthroscopic ACLR.

**Table 5:** Progression of osteoarthritis in the non-operated compartment.

Variable	Overall, n (%)	Primary ACLR, n (%)	Revision ACLR, n (%)	*p*
**Preoperative Kellgren–Lawrence**				
0	19 (67.9)	14 (50.0)	5 (17.9)	0.03
1	9 (32.1)	2 (7.1)	7 (25.0)	
**Last FU Kellgren–Lawrence**				
0	16 (57.1)	12 (42.9)	4 (14.3)	
1	12 (42.9)	4 (14.3)	8 (28.6)	

Note. ACLR = anterior cruciate ligament reconstruction; FU = follow-up; n = number. *p* value refers to differences in Kellgren–Lawrence distribution between the two groups.

## Data Availability

The original contributions presented in this study are included in the article. Further inquiries can be directed to the corresponding author.
